# Right Ventricular Dysfunction in Acute Respiratory Distress Syndrome and Its Quantification by Tricuspid Annular Plane Systolic Excursion on Transthoracic Echocardiography

**DOI:** 10.7759/cureus.76868

**Published:** 2025-01-03

**Authors:** Himanshu Satapathy, Asif Ahmed, Sujeet A Joshi, Lalit Sehgal, Sanjib K Dhar

**Affiliations:** 1 Critical Care Medicine, Sum Ultimate Medicare, Bhubaneswar, IND; 2 Critical Care Medicine, Tata Main Hospital, Jamshedpur, IND; 3 Liver Transplant Anaesthesia and Liver ICU, Shalby Sanar International Hospitals, Gurugram, IND; 4 Critical Care Medicine, BLK-Max Super Speciality Hospital, New Delhi, IND

**Keywords:** acute respiratory distress syndrome, echocardiography, mechanical ventilation, plasma bnp, right ventricular failure, tapse

## Abstract

Background

Right ventricular (RV) dysfunction is a typical complication of acute respiratory distress syndrome (ARDS), which is an independent predictor of poor prognosis in ARDS. Thus, evaluation of RV function is a crucial component of ARDS patient management. The present study aimed to determine the incidence of RV dysfunction by 2D echocardiography in mechanically ventilated ARDS patients in the ICU and assess the serial changes in tricuspid annular plane systolic excursion (TAPSE) among these patients in the intensive care unit (ICU) of a tertiary care cancer institute of Eastern India.

Methods

The prospective observational study included 40 patients aged 18-80 years who were admitted to the critical care unit with ARDS and put on mechanical ventilation. Patients meeting eligibility criteria underwent routine investigations on admission to the ICU. Key parameters included recording of the partial pressure of oxygen/fraction of inspired oxygen (PaO_2_/FiO_2_) oxygenation index, TAPSE on 2D transthoracic echocardiography, and plasma B-type (or brain) natriuretic peptide (BNP) level estimation conducted on day 0, day 2, and day 5. A TAPSE value <17 mm was considered to indicate RV dysfunction. Weaning outcomes, ventilator days, length of ICU stay, length of hospital stay, and in-ICU mortality were noted.

Results

Among the 40 study participants, the mean (± SD) age was 52.6 (± 14.3) years, and 52.5% were male patients. The incidence of RV dysfunction in ARDS was 17.5%. These patients were observed to have a progressive worsening of hypoxia along with a significant elevation of plasma BNP levels on day 2 and day 5 as compared to baseline. Weaning outcomes, ventilator days, length of hospital stay, and in-ICU mortality were comparable between the two groups.

Conclusion

The present study reports a 17.5% incidence of RV dysfunction in ARDS and quantifies a longitudinal deterioration in RV function with the onset of ARDS using TAPSE. The inclusion of objective indices on two-dimensional echocardiography, such as TAPSE, facilitates their regular application at the bedside and equips clinicians with a means to detect and quantify RV dysfunction in its early stages in ARDS.

## Introduction

Acute respiratory distress syndrome (ARDS) is a life-threatening condition characterised by hypoxaemia, non-cardiogenic pulmonary oedema, pulmonary inflammation, and decreased pulmonary compliance [1]. Despite the greatest efforts of the treating doctors, ARDS-related mortality rates still range between 30% and 40% [2,3]. Haemodynamic support and mechanical breathing are the mainstays of current ARDS therapy [4]. Right ventricular (RV) dysfunction is a typical complication of ARDS [5,6], which, along with pulmonary hypertension, are independent indicators of mortality linked to worse outcomes in ARDS patients [7-11]. Thus, evaluation of RV function is a crucial component of ARDS patient management [12].

The majority of research on RV dysfunction in ARDS uses invasive techniques, such as transesophageal echocardiography (TEE) and pulmonary artery catheters (PACs) [7-11]. In the current scenario, there has been a significant decline in the use of PACs in the management of patients with ARDS and a lack of availability and competence in TEE in the majority of intensive care units (ICUs) in India. Cardiac MRI, which is now the gold standard imaging method for RV evaluation, enables extremely accurate and reliable measurement of RV ejection fraction [13]. However, it is usually not possible to do an MRI on a severely ill patient. In these cases, echocardiography is useful since it may be done at the patient's bedside in a safer setting [14,15]. A non-invasive and economical screening method for ARDS patients is transthoracic echocardiography (TTE), which can identify elevated pulmonary vascular resistance and RV dysfunction. Recent additions to the toolbox for TTE RV function assessment include the tricuspid annular plane systolic excursion (TAPSE), RV fractional area change (RV-FAC), and the Tei index [16]. Earlier TTE investigations on ARDS patients were restricted to measuring RV size and function and systolic pulmonary artery pressure (SPAP) [17]. Of all the aforementioned parameters, TAPSE is a parameter of the global RV function that describes apex-to-base shortening and correlates closely with the right ventricular ejection fraction (RVEF) [18]. The proclivity towards using TAPSE for assessing RV systolic function is due to its ease of application and high reproducibility [19]. Moreover, TAPSE measurements showed excellent intra-observer reliability and inter-observer reliability [20].

Because of constraints in acoustic windows and the intricate design of the RV chamber, TTE assessment of RV function in patients with ARDS remains essentially qualitative despite recommendations by the American Society of Echocardiography (ASE) 2015 Chamber Guidelines [14] to include quantitative measures of RV performance [21,22]. There is limited evidence of their utility in the diagnosis of RV dysfunction and demonstrating change over time in patients with ARDS. Thus, the present study aimed to determine the incidence of RV dysfunction by two-dimension echocardiography in mechanically ventilated ARDS patients in the ICU and assess the serial changes in TAPSE among the study participants.

## Materials and methods

The present prospective observational study was conducted in the Department of Critical Care Medicine, Tata Main Hospital (TMH), Jamshedpur (a tertiary care hospital in Eastern India) from August 2021 to December 2022 after approval from the Institutional Ethics Committee, TMH, Jamshedpur (vide reference no. DMH/280/2021 dated 06.08.2021).

Study participants

The study included 40 patients aged between 18 and 80 years admitted to the critical care unit who met the Berlin definition of ARDS and were put on ventilatory support [23]. The exclusion criteria were as follows: patients with a history of chronic respiratory illness (long-term oxygen therapy or non-invasive ventilation); chronic right ventricular failure; chronic heart failure with left ventricular ejection fraction (LVEF) < 35%; severe valvular heart disease; in whom extracorporeal membrane oxygenation was started before echocardiography assessment. Patients who were discharged against medical advice and whose legal attendants did not provide informed written consent were excluded from the study.

Study procedure

Data collection was initiated after obtaining approval from the Institutional Ethics Committee of the concerned hospital. Upon admission to the ICU, all patients underwent severity assessment using Sequential Organ Failure Assessment (SOFA) and Acute Physiology and Chronic Health Evaluation (APACHE) scoring systems. Baseline demographics, including age, sex, primary and secondary aetiology of ARDS (pneumonia, aspiration, sepsis), and comorbid conditions, were collected on enrolment. The partial pressure of oxygen/fraction of inspired oxygen (PaO_2_/FiO_2_) oxygenation index, vasopressor requirement, ventilator parameters (plateau airway pressure, mean airway pressure, positive end-expiratory pressure (PEEP), tidal volume, respiratory system compliance, driving pressure), and mode of ventilation (volume control, pressure control, pressure support) were collected on enrolment and on day 2 and day 5.

The 2D transthoracic echocardiography was conducted on day 0, day 2, and day 5. Tricuspid annular plane systolic excursion (TAPSE) was measured as “the systolic displacement of the lateral portion of the tricuspid annulus during systole (M-mode)” [14]. A TAPSE value <17 mm was considered to indicate RV dysfunction [14]. Each measure was performed three times, and the mean value was recorded. All echocardiography findings were cross-checked and verified by a cardiologist. Plasma B-type (or brain) natriuretic peptide (BNP) level was also measured on day 0, day 2, and day 5. Weaning outcomes, ventilator days, length of ICU stay, length of hospital stay, and in-ICU mortality were noted.

Data analysis

Data collected was collated and entered in Microsoft Excel 2016 (Microsoft Corp., Redmond, WA, USA), and statistical analysis was done using the software Statistical Package for Social Sciences (SPSS) version 24.0 (IBM Corp., Armonk, NY, USA). The data was analysed using appropriate statistical tools and represented by various tables, graphs, diagrams, etc. Continuous variables were expressed as mean ± standard deviation (SD), and categorical variables were expressed as relative frequency and percentage. The association of incidence of RV dysfunction with demographic characteristics and baseline laboratory parameters was done through the chi-square test (for categorical variables) and independent sample t-test (for continuous variables). Repeated measures analysis of variance (ANOVA) test was used to find the association of the incidence of RV dysfunction with serial readings of the PaO_2_/FiO_2_ ratio and TAPSE on day 0, day 2, and day 5. P-value < 0.05 was considered statistically significant.

## Results

Baseline characteristics

Among the 40 patients who participated in the study, the mean (± SD) age was 52.6 (± 14.3) years, with the majority of the participants in the age group of 51-60 years (n=10, 25%), with 52.5% (n=21) of the participants male patients. Among 40 patients, 31 (77.5%) had one or more comorbidities, which included hypertension (n=21, 52.5%), diabetes (n=19, 47.5%), and hypothyroidism (n=3, 7.5%). The most frequent cause of ARDS was pneumonia, reported in 37.5% of the study participants (n=15), followed by pancreatitis (n=10, 25%), liver diseases (n=5, 12.5%), and sepsis (n=4, 10%). Less common reported causes were road traffic accidents (RTA) polytrauma, CA ovary (ovarian cancer), and poisoning (5% each) (Table 1).

**Table 1:** Distribution of study participants according to primary diagnosis (N=40). *Includes acute liver failure (n=1), chronic liver disease (n=2), decompensated liver disease (n=1) and acute on chronic liver disease (n=1); **Includes sepsis (n=2), puerperal sepsis (n=1) and urosepsis (n=1); ^Includes organophosphorus poisoning (n=1) and aluminium phosphide poisoning (n=1). RTA polytrauma: road traffic accidents polytrauma, CA ovary: ovarian cancer.

Primary diagnosis	Frequency n (%)
Pneumonia	15 (37.5)
Pancreatitis	10 (25.0)
Liver disease*	5 (12.5)
Sepsis**	4 (10.0)
RTA polytrauma	2 (5.0)
CA ovary	2 (5.0)
Poisoning^	2 (5.0)

Incidence of RV dysfunction

On admission, the mean (± SD) APACHE II score was 14.7 (± 65.1), and the SOFA score was 7.7 (± 1.9). Of the 40 patients, 22 required vasopressors (55%). A total of 7 patients developed RV dysfunction (three cases within 48 hours of MV and four patients between day 2 and day 5), accounting for an incidence of 17.5% among patients with ARDS requiring mechanical ventilation.

Factors associated with RV dysfunction

The mean ± SD age was 53.2 ± 6.2 years among those who developed RV dysfunction and 49.8 ± 10.1 years among those who did not develop the same; however, this difference was not statistically significant (p = 0.743). The patients in both groups were also comparable in terms of gender distribution, presence of comorbidities, and baseline blood parameters measured at the time of admission to the ICU (p > 0.05).

In the present study, a progressive worsening of hypoxia (marked by a temporal decrease in the mean PaO_2_/FiO_2_ ratio from day 0 to day 5) was noted among the seven patients who developed RV dysfunction, which was statistically significant (p < 0.001). On the contrary, the remaining 33 patients were observed to have a progressive improvement in hypoxia (marked by a temporal increase in the mean PaO_2_/FiO_2_ ratio from day 0 to day 5) (p < 0.001) (Table 2).

**Table 2:** Comparison of severity of hypoxia assessed by PaO2/FiO2 ratio on day 0, day 2 and day 5 between study participants with and without RV dysfunction (N=40). Values are presented as mean ± SD. *Based on Mann-Whitney U test (non-parametric); #Based on Kruskal-Wallis test (non-parametric); P<0.05 is considered as statistically significant. PaO_2_/FiO_2_: partial pressure of oxygen/fraction of inspired oxygen; RV: right ventricular.

Study groups	PaO_2_/FiO_2_ ratio			p-value^#^
	Day 0	Day 2	Day 5	
Developed RV dysfunction	122.3 ± 27.9	112.6 ± 21.4	103.1 ± 25.5	<0.001
Did not develop RV dysfunction	125.2 ± 35.4	131.4 ± 33.9	137.5 ± 36.3	<0.001
p-value*	0.526	0.021	<0.001	

TAPSE and RV dysfunction

Among the patients with RV dysfunction, the mean TAPSE value on day 0 was 22.4 ± 3.6 mm, while the figure was 16.2 ± 1.9 mm on day 2 and 15.3 ± 1.3 mm on day 5. Notably, there was a significant reduction in TAPSE values on day 2 and day 5 when compared to baseline (p=0.037). Moreover, TAPSE values on day 0, day 2, and day 5 of the patients who developed RV dysfunction were significantly lower than those of the patients who did not develop RV dysfunction on the corresponding day (p < 0.05) (Table 3).

**Table 3:** Comparison of serial values of TAPSE on 2D echocardiography measured on day 0, day 2 and day 5 between study participants with and without RV dysfunction (N=40). Values are presented as mean ± SD. *Based on Mann-Whitney U test (non-parametric); #Based on Kruskal-Wallis test (non-parametric); P<0.05 is considered as statistically significant. TAPSE: tricuspid annular plane systolic excursion; RV: right ventricular.

Study groups	TAPSE (mm)			p-value^#^
	Day 0	Day 2	Day 5	
Developed RV dysfunction	22.4 ± 3.6	16.2 ± 1.9	15.3 ± 1.3	0.037
Did not develop RV dysfunction	26.5 ± 5.8	22.5 ± 3.8	24.7 ± 4.9	0.206
p-value*	0.046	0.003	<0.001	

Plasma BNP and RV dysfunction

Patients with RV dysfunction also demonstrated a significant elevation in plasma BNP levels on day 2 and day 5 when compared to baseline (p = 0.002). On day 0, BNP levels were comparable between the patients with and without RV dysfunction (p = 0.217). However, on day 2 and day 5, BNP levels were significantly raised among the patients who developed RV dysfunction as compared to those who did not (p < 0.001) (Table 4).

**Table 4:** Comparison of serial values of plasma BNP levels measured on day 0, day 2 and day 5 between study participants with and without RV dysfunction (N=40). Values are presented as mean ± SD. *Based on Mann-Whitney U test (non-parametric); #Based on Kruskal-Wallis test (non-parametric); P<0.05 is considered as statistically significant. BNP: B-type natriuretic peptide; RV: right ventricular.

Study groups	Plasma BNP (pg/ml)			p-value^#^
	Day 0	Day 2	Day 5	
Developed RV dysfunction	79.9 ± 19.4	337.2 ± 38.6	386.3 ± 74.8	0.002
Did not develop RV dysfunction	76.5 ± 21.8	99.2 ± 37.9	84.3 ± 26.0	0.639
p-value*	0.217	<0.001	<0.001	

Outcome parameters and RV dysfunction

Weaning outcomes could be determined only for those patients in whom weaning attempts were made. Weaning success rates were 75% in the first group and 77.8% in the latter group, with no statistical difference between the two proportions. The mean ± SD ventilator days were 14.3 ± 3.7 days among those who developed RV dysfunction and 11.6 ± 5.1 days among those who did not develop RV dysfunction; however, this difference was not statistically significant (p = 0.239). The patients in both groups were also comparable in terms of length of ICU stay (p = 0.823) and length of hospital stay (p = 0.953). Overall, out of the 40 study participants, 15 patients expired, accounting for a mortality rate of 37.5% in ARDS patients on mechanical ventilation. On stratified analysis, the mortality rates were 42.9% among those with RV dysfunction and 36.4% among those who did not develop RV dysfunction, with no significant difference (p = 0.747) (Table 5).

**Table 5:** Comparison of outcome parameters between study participants who developed and did not develop RV dysfunction (N=40) Values are presented as n (%) or mean ± SD. #Based on the Chi-square test for categorical data and the Mann-Whitney U test (non-parametric) for continuous data; P<0.05 is considered as statistically significant. RV: right ventricular.

Parameters	Developed RV dysfunction (N=7)	Did not develop RV dysfunction (N=33)	p-value^#^
Weaning attempts			
0	3 (42.9)	6 (18.2)	0.554
1	1 (14.2)	7 (21.2)	
2	3 (42.9)	15 (45.4)	
3	0	5 (15.2)	
Weaning outcome (n=31)			
Success	3 (75.0)	21 (77.8)	0.901
Failure	1 (25.0)	6 (22.2)	
Ventilator days	14.3 ± 3.7	11.6 ± 5.1	0.239
Length of ICU stay	15.4 ± 4.2	14.9 ± 4.4	0.823
Length of hospital stay	18.3 ± 4.9	19.1 ± 4.8	0.953
Patient outcome			
Discharged	4 (57.1)	21 (63.6)	0.747
Expired	3 (42.9)	12 (36.4)	

## Discussion

The most severe type of acute lung damage, known as acute respiratory distress syndrome (ARDS), is characterised by acute bilateral pulmonary infiltrates, decreased lung compliance, and moderate to severe hypoxaemia [24]. RV dysfunction with accompanying haemodynamic impairment, in particular, is becoming more widely recognised as a predictor of a poor clinical prognosis in ARDS patients [6,25]. The American Society of Echocardiography has recently advocated the use of quantitative indices for assessing RV function on TTE due to a lack of competence in TEE and a decrease in the use of PACs [14].

In the present study, RV dysfunction, or more specifically RV systolic impairment, was defined as a reduction in the TAPSE to values less than 17 mm on 2D echocardiography [14,26]. According to the pre-decided study methodology, transthoracic echocardiography conducted on all patients on the day of admission ruled out the onset of RV dysfunction prior to admission to the ICU among the study participants. Additionally, the mean ± SD BNP level on the day of admission was 77.2 ± 20.9 pg/ml, supporting the absence of cardiogenic pathology in such patients on the day of ICU admission. The mean ± SD TAPSE value on the day of admission was 21.5 ± 4.8 mm. Out of the 40 patients, there were 4 patients who developed RV dysfunction on day 2, and 3 patients were found to develop RV dysfunction on day 5. Overall, the incidence of RV dysfunction among our study participants was 17.5%. This finding is similar to the 21% incidence reported by Sato et al. in 2021 in their systematic review and meta-analysis [27].

A new door has been opened in the treatment of ARDS via echocardiography. Although the pathophysiological mechanism(s) or clinical impact of these alterations have not yet been fully understood, it is still possible to detect various morphological and functional changes in the right ventricle in these patients [28]. In patients with ARDS, the pathogenesis of RV damage is complicated, but it is predominantly caused by a rise in pulmonary vascular resistance brought on by persistent inflammation, hypoxaemia-induced vasoconstriction, microthrombi development, and vascular remodelling [29]. Acute cor pulmonale develops in these patients as a result of the right ventricle's thin walls, insufficient contractile reserve, and inability to respond appropriately to a rapid increase in afterload. Positive pressure breathing increases RV afterload from increased intrathoracic pressure, which worsens RV damage in patients with ARDS [30].

The echocardiographic parameter TAPSE evaluates the reduction in length from the base to the apex of the right ventricle and signifies the longitudinal contraction of the ventricle. TAPSE is a direct evaluation of right ventricular function and has been deemed appropriate for detecting early alterations in right ventricular physiology in ARDS [31]. This study revealed that patients experienced acute RV dysfunctional changes following the onset of ARDS. We successfully recorded the longitudinal deterioration in the function of the right ventricle with the onset and progression of ARDS by utilising the objective marker of TAPSE on TTE. Further, RV function demonstrated a correlation with ARDS severity (given by the PaO_2_/FiO_2_ ratio), as indicated by the progressively worsening hypoxia from day 0 to day 5.

In terms of various outcome parameters noted in our study, weaning success rates, ventilator days, length of ICU stay, and length of hospital stay were comparable between patients who developed RV dysfunction in ARDS and those who did not develop the same. Moreover, although the mortality among patients with RV dysfunction was numerically higher than in the non-RV dysfunction group, the difference did not achieve statistical significance among our study participants. This finding is similar to that reported by Fichet et al. but is in contrast to a positive association between RV function and mortality reported by Wadia et al. [32,33].

The present study also considered measurements of plasma BNP on day 0, day 2, and day 5 in concordance with transthoracic echocardiography at the corresponding time points. BNP has been identified as a reliable marker in various heart conditions. The heart ventricles release BNP as a result of greater mechanical stress and distension of the heart wall. BNP shields the heart from negative effects caused by excessive strain by enhancing the excretion of sodium and causing diuresis, relaxing the smooth muscles of the blood vessels, suppressing the renin-angiotensin-aldosterone system, and cardiac remodelling through hypertrophy and fibrosis [34]. We observed a significant rise in plasma BNP as compared to baseline values among the patients with ARDS who developed RV dysfunction, which can be attributed to the stretching of the right ventricular wall caused by pressure overload and impaired function of the right ventricle. However, it must also be kept in mind that raised BNP levels are also widely associated with myocardial infarction and left heart failure and are non-specific for RV dysfunction, thus demanding the treating physicians to rule out other cardiac pathologies before affirming the diagnosis of RV dysfunction based on raised plasma BNP levels.

The study had the following limitations. First, only TAPSE on 2D echocardiography was measured. The use of other objective parameters on echocardiography, such as right ventricular myocardial performance index (MPI), right ventricular fractional area change (FAC), systolic pulmonary artery pressure (SPAP), peak tricuspid regurgitation (TR) velocity, and presence or absence of septal shift, would have helped generate a comprehensive understanding of the pathological changes in the heart in ARDS. Secondly, the research was conducted in a single hospital, and the findings of the study may not be valid beyond the present study setting. Lastly, the current research is limited by a small sample size. Further large-scale multicentric studies are warranted to validate the findings of the present work.

## Conclusions

To the best of our knowledge, this is the only study conducted in an Indian ICU setting to determine the incidence of right ventricular dysfunction in patients with ARDS requiring mechanical ventilation and assess the serial changes in TAPSE by 2D echocardiography in such patients. The incidence of RV dysfunction in our study was found to be 17.5%. Also, the present study was able to quantify longitudinal deterioration in RV function with the onset of ARDS using the objective marker of TAPSE on TTE. The current recommendations for improving outcomes in ARDS include a “RV-protective” management strategy along with a “lung-protective” strategy. The inclusion of objective indices on two-dimensional echocardiography, such as TAPSE, facilitates their regular application at the bedside and equips clinicians with a means to detect and quantify RV dysfunction in its early stages in ARDS.
